# Gender Differences in Post-Traumatic Stress

**DOI:** 10.1089/biores.2017.0004

**Published:** 2017-02-01

**Authors:** Maria Grazia Modena, Daniele Pettorelli, Giulia Lauria, Elisa Giubertoni, Erminio Mauro, Valentina Martinotti

**Affiliations:** ^1^ Dipartimento Chirurgico, Medico, Odontoiatrico e di Scienze Morfologiche con interesse Trapiantologico, Oncologico e di Medicina Rigenerativa, UNIMORE, Modena, Italy.; ^2^ Scuola di Specializzazione in Malattie Cardiovascolari Azienda Ospedaliera Universitaria Policlinico, Modena, Italy.; ^3^ Dipartimento Emergenza-Urgenza, UNIMORE, Modena, Italy.

**Keywords:** cardiovascular disease in women, earthquake, gender, stress

## Abstract

Acute stress can trigger cardiovascular events and disease. The earthquake is an “ideal” natural experiment for acute and chronic stress, with impact mainly on the cardiovascular system. On May 20th and 29th, 2012, two earthquakes of magnitude 5.9° to 6.4° on the Richter scale, hit the province of Modena and Reggio Emilia, an area of the north-center of Italy never considered at seismic risk. The purpose of our study was to assess whether there were gender-specific differences in stress-induced incidence of cardiovascular events and age of patients who arrived at the Emergency Departments (ED) of the three main teaching hospitals of the University of Modena and Reggio Emilia. Global access of patients, divided in relation to age, gender, and diagnosis was compared with that one detected in the same departments and in the same interval of time in 2010. The data collected were relative to consecutive cases derived by retrospective chart and acute cardiovascular events were classified according to ICD-9 (*International Classification of Diseases*, ninth revision). A total of 1,401 accesses were recorded in the year of earthquake versus 530 in 2010 (*p* ≤ 0.05), with no statistically significant differences in number of cases and mean age in relation to gender, despite the number of women exceeded that of men in 2012 (730 vs. 671); the opposite occurred, in 2010 (328 vs. 202). The gender analysis of 2012 showed a prevalence of acute coronary syndromes (ACSs 177 vs. 73, *p* ≤ 0.03) in men, whereas women presented more strokes and transient ischemic attacks (TIAs) (90 vs. 94, *p* ≤ 0.05), atrial fibrillation (120 vs. 49, *p* ≤ 0.05), deep venous thrombosis and pulmonary embolism (DVT/PE; 64 vs. 9, *p* ≤ 0.05), panic attacks (124 vs. 26, *p* ≤ 0.03), aspecific chest pain (122 vs. 18, *p* ≤ 0.05), TakoTsubo cardiomyopathy (10 vs. 0, *p* ≤ 0.05), and DVT/PE (61 vs. 3, *p* ≤ 0.03). The gender analysis of 2010 showed no difference in number of accesses and age, with higher incidence of ACS in men (130 vs. 34, *p* ≤ 0.05) and aspecific chest pain in women (42 vs. 5, *p* ≤ 0.05). The analysis between 2012 and the standard period (2010) showed women recurring to ED in larger number with more panic attacks (124 vs. 3, *p* ≤ 0.01), more atrial fibrillation (120 vs. 40, *p* ≤ 0.01) and, as a possible consequence, more TIAs and strokes (190 vs. 25, *p* ≤ 0.005), more TakoTsubo (10 vs. 0, *p* ≤ 0.05), DVT/PE (61 vs. 3, *p* ≤ 0.05), and aspecific chest pain (122 vs. 5, *p* ≤ 0.01). The difference between men's accesses to ED was less striking, but in 2012 men reported more panic attacks (26 vs. none, *p* ≤ 0.05), more atrial fibrillations, TIAs, and strokes (49 vs. 13, *p* ≤ 0.05 and 94 vs. 18, *p* ≤ 0.03). In conclusion, clinical (stress induced) events recorded during and immediately after the 2012 earthquakes were quite different between women and men, although the pathophysiological mechanism was probably the same, consisting acute sympathetic nervous activation, with elevation of blood pressure and heart rate, endothelial dysfunction, platelet and hemostatic activation, increased blood viscosity, and hypercoagulation. Women, in our observation, appeared to be more sensitive and responsive to acute stress, although men also appeared to suffer from stress effects when compared with a standard period, which, nevertheless, reflects in our population the most common epidemiology of gender difference in ED accesses for cardiovascular events.

## Introduction

Population research provides a unique opportunity to gather information that allows comparison of both traditional and novel cardiovascular risk factors by sex. Among novel gender-related risk factors, psychosocial issues such as depression, abuse, and domestic violence, and, in particular, post-traumatic stress disorders are of growing interest.^1^

We focus in this report on post-traumatic stress disorders related to cardiovascular event as the consequence of the earthquake, which occurred in 2012 in our region, an area never considered at seismic risk. For this reason, despite the fact that the earthquake was not so severe as to cause deadly consequences, it was the shock of an unexpected natural disaster that induced cardiovascular events. Some individuals are hyperresponsive to the activation of sympathetic nervous system after acute psychological stimuli and current data link sympathetic nervous system hyperresponsivity to be a trigger and to exacerbate coronary and vascular atherosclerosis.

On May 20th and 29th, 2012, there were two earthquakes of magnitude 5.9° to 6.4° on the Richter scale, which hit 33 municipalities of the province of Modena and Reggio Emilia.

**FIG. 1.**
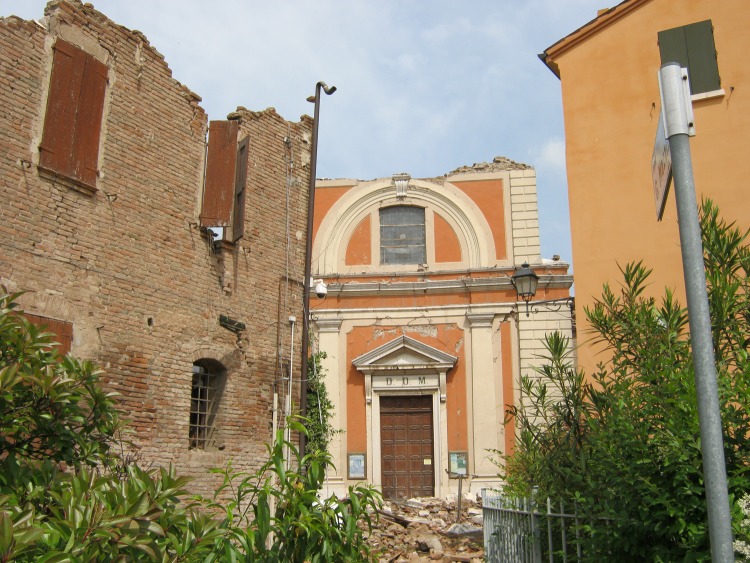
Parish Church of XVII century destroyed by the earthquake. City of San Felice (Modena).

In all these municipalities, destruction of city centers with different levels of severity occurred. In the province of Modena and Reggio Emilia, according to a classification made by the civil defense, the different cities and villages reported a grade of gravity from 9 to 6. These events caused heavy damage to rural and industrial buildings, to channeling of water, as well as to buildings and historical monuments of artistic interest (Fig. 1) and to the civil old buildings in stone or pebbles. Around 270 schools were totally or partially unusable; there were damages to residential use of new buildings, often attributable to widespread incidents of soil liquefaction. According to an account of the Civil Protection who checked 35,000 structures in Emilia, 22.5% of the buildings were found temporarily or partially unusable, 35.7% and 5.7% unusable for external risk due to elements unsafe whose collapse could affect the building. More than 28 reception areas that received about 5800 people were activated. The total number of people housed was found to be 8200. Unexpected events and conditions just described have created stress, loss, and fear in affected populations, with important impacts on the state of physical and mental health.

Several studies have shown that environmental disasters lead to an increase in cardiovascular diseases. Most published studies, to our knowledge, focus on the assessment of the general population. Studies that take into account gender differences in cardiovascular events related to earthquakes and disasters are very limited.^2–4^

### Objective of the study

The purpose of our study was to assess whether there was gender difference in stress-induced incidence and presentation of cardiovascular events related to earthquakes of May 20th and 29th, 2012 in Emilia a large, rich region in north-center of Italy. Hospital records could only be collected after the events because of damage to the hospitals' structures.

### Inclusion criteria

• Men and women over 18 years.
• Cardiovascular events due to acute coronary syndrome (ACS; unstable angina [UA], non-ST elevation and ST elevation myocardial infarction [STEMI], non-STEMI and STEMI, cardiac arrest), exacerbation of heart failure, arrhythmias, stress cardiomyopathy (TakoTsubo Syndrome), transient ischemic attacks (TIA), stroke, thromboembolic events (deep venous thrombosis and pulmonary embolization [DVT/PE]), arrhythmias, and hypertensive crisis.
• Noncardiovascular events (aspecific chest pains, panic attacks).

## Materials and Methods

We enrolled patients referred to the Emergency Departments (ED) of the three main hospitals of the cities of Modena (NOCSAE—an acronymous for Baggiovara Hospital—and the Policlinico) and Reggio Emilia (ASMN—Azienda Santa Maria Nuova Hospital) in the period between May 20th and June 13th, 2012. All three hospitals are teaching hospitals of the University of Modena and Reggio Emilia. The total access of patients was compared with that recorded in the same departments and in the same period of time of year 2010. We had to restrict the evaluation to 3 weeks because data collection was limited by the postearthquake renovation of the most damaged hospital (Policlinico). We did not obtain the informed consent from patients because our study was a retrospective analysis of hospitals records and it was not possible, unfortunately, to start a survey on cardiovascular risk and events. Acute cardiovascular events were classified according to ICD-9 (*International Classification of Diseases*, ninth revision) and ACS diagnosis confirmed by pain, enzymes and/or electrocardiographic changes.

### Data analysis

Collected data were relative to consecutive cases derived by retrospective chart, with IRB approval and validation of oversight of the Responsible Conduct of Research and were analyzed as means and standard deviations for continuous variables and as percentages for categorical frequencies. Results were processed in total and stratified by age and sex. Continuous variables normally distributed were compared using the Student's *t*-test for independent samples. A *p*-value <0.05 was considered statistically significant. All data were age-standardized using the standard European population so that it was possible to make comparisons with the results of the number of access in the three ED in the same period of year 2010. The design of the study, preliminary, partial, results, and ethics committee permission, have been all published in Italy in a volume where the Rector of University of Modena and Reggio Emilia decided to collect all research contributions carried out on the earthquake of 2012.^5^

## Results

A total of 1401 accesses were recorded in the period of earthquakes with an increase of 45% in comparison to the same time of observation of 2010. No statistically significant difference was observed in mean age and number of accesses in relation to gender, although women were more (730 vs. 671 men), whereas causes of hospitalization were quite different between sexes (Table 1). Comparison in gender causes of access showed women to report more panic attacks (124 vs. 26, *p* ≤ 0.03), aspecific chest pain (122 vs. 18, *p* ≤ 0.05), and atrial fibrillation (120 vs. 49, *p* ≤ 0.05) as the most frequent arrhythmias in both genders and, as possible consequence, more TIAs and strokes (190 vs. 94, *p* ≤ 0.05). Men reported more ACS (177 vs. 78, *p* ≤ 0.03) either STEMI or non-STEMI, whereas women presented UA/non-STEMI and TakoTsubo cardiomyopathy (10 vs. none, *p* ≤ 0.05). Gender analysis of later cause of accesses showed a prevalence of DVT/PE in women (64 vs. 9, *p* ≤ 0.05), recorded 1 or 2 weeks from the seismic shakes and attributable to the long stay in the car in the sitting position, which facilitates the formation of thrombosis of lower limbs veins. Most people in fact, in those days, for fear, slept in the car. Ventricular arrhythmias were less than atrial fibrillation, probably because they are among main causes of sudden death outside the hospital. On extra-hospital sudden deaths the competence is of the Coroner Department; we do not therefore have any data. Other external causes of deaths were reported as consequence of buildings collapse. The gender analysis of 2010 (Table 2) showed no difference in number of accesses and age, with higher incidence of ACS in men (130 vs. 34, *p* ≤ 0.05) and aspecific chest pain in women (42 vs. 5, *p* ≤ 0.05). Finally, comparison between 2012 and a standard period (2010) showed (Table 3) women recurring to ED in larger number with more panic attacks (124 vs. 3, *p* ≤ 0.01), more atrial fibrillation (120 vs. 40, *p* ≤ 0.01) and, as a possible consequence, more TIAs and strokes (190 vs. 25, *p* ≤ 0.005), TakoTsubo (10 vs. 0, *p* ≤ 0.05), DVT/PE (61 vs. 3, *p* ≤ 0.05), and aspecific chest pain (122 vs. 5, *p* ≤ 0.01). The difference between men's accesses to ED was less striking, but in 2012 men reported more panic attacks (26 vs. none, *p* ≤ 0.05), more atrial fibrillations, TIAs, and strokes (49 vs. 13, *p* ≤ 0.05 and 94 vs. 18, *p* ≤ 0.03). The choice of 2010 has been done because Departmental structures were comparable to the year of earthquake (some changes occurred in 2011, moving back in 2012).

**Table 1.** **Study Group**

Number of accesses to University Hospitals divided in relation to gender and RCR-derived diagnosis: 2012				
2012	Men, age 58; SD 21.8	Age	Women, age 60.9, SD 22.7	Age
Total accesses	671		730	
Panic attacks, *n* (%)	26 (3.8)	58 ± 9.5	124 (16.9)	60 ± 21.6
Arrhythmias	90 (13.4%; 55% AF)	58 ± 19.2	160 (21.9%; 75% AF)	60 ± 16.4
TIA/stroke, *n* (%)	94 (14)	69 ± 9.5	190 (26)	70 ± 10.8
ACS	177 (more NSTEMI and STEMI)	58 ± 12.5	78 (more UA/NSTEMI)	69 ± 11
DVT/PE, *n* (%)	9 (1.34)	59 ± 20	64 (8.76)	61 ± 15.4
Acute HF, *n* (%)	84 (12.5)	65 ± 12.5	66 (9)	65 ± 21.4
Hypertensive crisis, *n* (%)	28 (4.1)	67 ± 10.6	66 (9)	68 ± 9.8
TakoTsubo, *n* (%)	0		10 (1.36)	67 ± 2.9
Chest pain, *n* (%)	18 (2.6)	59 ± 18.6	122 (16.7)	60 ± 21.6

Comparison between men and women in relation to diagnosis and cause of access, 2012			
2012	Men, age 58; SD 21.8	ns	Women, age 60.9, SD 22.7
Total accesses	671	ns	730
Panic attacks	26	*p* ≤ 0.03	124
Arrhythmias	90	*p* ≤ 0.05	160
AF	49	*p* ≤ 0.05	120
TIA/stroke	94	*p* ≤ 0.05	190
ACS	177 (more NSTEMI and STEMI)	*p* ≤ 0.03	78 (more UA/NSTEMI)
DVT/PE	9	*p* ≤ 0.05	64
Acute HF	84	ns	66
Hypertensive crisis	28	ns	66
TakoTsubo	0	*p* ≤ 0.05	10
Chest pain	18	*p* ≤ 0.05	122

ACS, acute coronary syndrome; acute HF, acute heart failure; AF, atrial fibrillation; DVT/PE, deep venous thrombosis and pulmonary embolism; NSTEMI, non-ST elevation myocardial infarction; RCR, Responsible Conduct of Research; SD, standard deviation; STEMI, ST elevation myocardial infarction; TIA, transient ischemic attack; UA, unstable angina.

**Table 2.** **Control Group**

Number of accesses to the University Hospitals in relation to gender and RCR-derived diagnosis: 2010				
2010	Men, age 60; SD 15.9	Age	Women, age 66; SD 20.5	Age
Total accesses	328		202	
Panic attacks, *n* (%)	0		3 (1.4)	60 ± 5
Arrhythmias, *n* (%)	68 (20 AF)	59 ± 14.5	54 (75 AF)	64 ± 15.5
TIA/stroke, *n* (%)	18 (5.48)	67 ± 12.5	25 (12)	70 ± 8.5
ACS, *n* (%)	130 (39) (more NSTEMI)	55 ± 10.9	34 (16.8) (more UA/NSTEMI)	69 ± 9.5
DVT/PE, *n* (%)	2 (0.6)	61 ± 14.3	3 (1.4)	65 ± 19.7
Acute HF, *n* (%)	40 (12)	68 ± 8.9	43 (21)	69 ± 15.7
Hypertensive crisis, *n* (%)	28 (8.5)	67 ± 5.9	35 (17)	70 ± 6.6
TakoTsubo	0		0	
Chest pain, *n* (%)	42 (12.8)	60 ± 14.6	5 (2.4)	65 ± 20

Comparison between men and women in relation to diagnosis and cause of access, 2010			
2010	Men, age 60; SD 15.9	ns	Women, age 66; SD 20.5
Total accesses	328	ns	202
Panic attacks	0	ns	3
Arrhythmias	68	ns	54
AF	13	ns	40
TIA/stroke	18	ns	25
ACS	130 (more NSTEMI)	*p* ≤ 0.05	34 (more UA/NSTEMI)
DVT/PE	2	ns	3
Acute HF	40	ns	43
Hypertensive crisis	28	ns	35
TakoTsubo	0		0
Chest pain	42	*p* ≤ 0.05	5

**Table 3.** **Comparison in Accesses Between Women and Between Men in 2010 Versus 2012**

	Comparison accesses between 2010 and 2012: women			Comparison accesses between 2010 and 2012: men		
	2010, age 66 years; SD 20.5	ns	2012, age 60.9 years; SD 22.7	2010, age 60 years; SD 15.9	ns	2012, age 58 years; SD 21.8
Total accesses	202	*p* ≤ 0.01	730	328	*p* ≤ 0.03	671
Panic attacks	3; 60 ± 5	*p* ≤ 0.001	124; 60 ± 21.6	0	*p* ≤ 0.05	26; 58 ± 9.5
Arrhythmias	54; 64 ± 15.5	*p* ≤ 0.03	160; 60 ± 16.4	68; 59 ± 14.5	ns	90; 58 ± 19.2
AF	40	*p* ≤ 0.01	120	13; 68 ± 6	*p* ≤ 0.05	49; 59 ± 12
TIA/stroke	25; 70 ± 8.5	*p* ≤ 0.005	190; 70 ± 10.8	18; 67 ± 12.5	*p* ≤ 0.03	94; 69 ± 9.5
ACS	34; 69 ± 9.5	ns	78; 69 ± 11	130; 55 ± 15.9	ns	177; 58 ± 12.5
DVT/PE	3; 65 ± 19.7	*p* ≤ 0.03	64; 61 ± 15.4	2; 61 ± 14.3	ns	9; 59 ± 20
Acute HF	43; 69 ± 15.7	ns	66; 65 ± 21.4	40; 68 ± 8.9	*p* ≤ 0.05	84; 65 ± 12.5
Hypertensive crisis	35; 70 ± 6.6	ns	66; 68 ± 9.8	28; 67 ± 5.9	ns	28; 67 ± 10.6
TakoTsubo	0	*p* ≤ 0.05	10; 67 ± 2.9	0		0
Chest pain	5; 65 ± 20	*p* ≤ 0.001	122; 60 ± 21.6	42; 60 ± 14.6	ns	18; 59 ± 18.6

## Discussion

The earthquake is an “ideal” natural experiment of acute and chronic stress, which finds the cardiovascular system as an ideal target to “rage” during both acute and chronic phase.

**FIG. 2.**
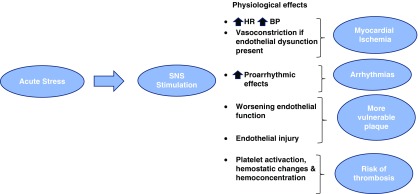
Acute stress effects: the stimulation of the SNS induces the increase of HR and BP, which, in turn, causes a number of alterations that can trigger cardiovascular events. BP, blood pressure; HR, heart rate; SNS, sympathetic nervous system.

Acute sympathetic nervous activation, stress-induced, is a risk factor that triggers cardiovascular events and progression of atherosclerosis through elevation of blood pressure and heart rate, endothelial cell dysfunction, platelet and hemostatic activation, increased blood viscosity, and increased imbalance between coagulation and fibrinolysis, with specific clinical consequences (Fig. 2).

Several studies have shown that environmental disasters lead to an increase in cardiovascular events and diseases. No study, to our knowledge, reported gender analysis in hospitals access after an earthquake. In the Athens earthquake, the Newcastle earthquake of Australia, and the Northridge earthquake of Los Angeles,^6–8^ the increase in cardiovascular deaths was limited to a few days. In the Hanshin-Awaji earthquake, the increase in cardiovascular deaths persisted on average 2 weeks, up to 1 month and more.^9^ All these studies investigated the effects of acute and subacute psychological stress caused by a sudden general disaster on mortality from atherosclerotic heart disease (underlying cause) and cardiac events (proximate cause) by comparing total and cause-specific mortality during the days after a major earthquake with the mortality during the surrounding month and the corresponding periods of different years. We followed a similar approach, but analyzing gender difference in causes of hospital access early and within 1 month after earthquake, comparing the same data, gender, and types with the corresponding period of year 2010. In our study, nine cases of cardiac arrest were detected, six of which were women. One peculiar aspect of our study is that the mean age of people of both genders arriving at the hospital ranged between 58 (SD 21.8) years for men and 60.9 (SD 22.7) years for women, mostly for stress-induced symptoms. Elderly and young people preferred not to leave home, or place of work, typical for a population of rural origins and habits. This makes our study different in results, because all prementioned reports were related to regions well known to be at high seismic risk, thus people are alerted and monitored, and buildings are constructed according to antiseismic regulations. Our region instead was never recognized as at seismic risk; many cities and villages are old with several historical monuments as well as many rural structures and only modern buildings are constructed according to specific regulations. All these reasons probably justify stress-induced effects to be dramatic and the reason for such a large effluence to Emergency Rooms/Hospitals. In other words, we observed more seismic-induced stress than seismic-induced mortality, because of a lower degree of severity of earthquake in our region.

In our report, number and mean age of women and men were similar, but types of events quite different. Women presented more atrial fibrillation, in relation to abrupt increase in blood pressure, and more TIAs and strokes in the first days. Atrial fibrillation is usually a nonfatal arrhythmia, but it justifies the high percentage of cerebrovascular complications such as stroke and, mostly, transient ischemic attacks. The pathophysiology is attributable to a sharp increase in systemic blood pressure from stress (hypertensive crisis were much more frequent in women), which causes an increase in left atrial pressure and volume, with onset of paroxysmal atrial fibrillation. In the absence of prompt administration of anticoagulant, as plausible in such a dramatic condition, atrial fibrillation may induce thrombus formation in the left atrium with systemic embolism.

In the following days, again more women presented with DVT/PE, related to a state of activation of coagulation, which takes more time to onset. In the Hanshin-Awaji earthquake^9^ in fact, increase in Von Willebrand factor and tissue plasminogen activator was observed after the earthquake in the high-stress hypertensive group, with a positive correlation between the levels of these two factors and D-dimer levels after the earthquake. We did not collect similar data, but this hypothesis is quite suggestive. Men presented more cases of ACS. In both genders there was a prevalence of non-STEMI, double in men (103 vs. 55), whereas STEMI were more present in men and all 10 cases of TakoTsubo syndrome were among women only, who presented also more noncardiac events such as panic attacks and aspecific chest pain, with normal electrocardiogram and without troponin elevation. Studies that take into account gender differences in cardiovascular events related to earthquakes and disasters are very limited.^2–4^ Gender means the complex interrelationship and integration of sex (to be understood as a biological and chromosomal marker) with the psychological and cultural attitude (due to ethnic, social, and religious affiliation). “Gender” is an important issue in health and especially in the cardiovascular field, where it has been shown that symptoms and pathophysiology of infarction, the most relevant event, are different between men and women.^10^ In particular, women may suffer a heart attack later than men, have more frequently microvascular angina, and present in almost an exclusive way a rare form of cardiomyopathy best defined by stress or TakoTsubo syndrome. It consists of heart attack as a result of strong emotional stress, from rush of adrenergic stimulation, and relay between the brain and the heart.^11^ The consequence is an apparent form of ACS with aneurysmal dilatation of the apex of the left ventricle, which takes the form of a flask or TakoTsubo, the container used in Japan (where cardiomyopathy was first described) to fish and trap octopus. Coronary arteries are usually angiographically normal and left ventricular dysfunction reversibile. Also, arrhythmias and acute thromboembolic episodes show gender differences.^12^ In conclusion, clinical (stress induced) events recorded during and immediately after the 2012 earthquake were quite different between women and men, although the pathophysiological mechanism was probably the same, consisting of acute sympathetic nervous activation, with elevation of blood pressure and heart rate, endothelial dysfunction, platelet and hemostaticactivation, increased blood viscosity, and hypercoagulation. Women, in our observation, appeared to be more sensitive and responsive to acute stress, although men also appeared to suffer from stress effects when compared with a standard period; nevertheless, this reflects in our population the most common epidemiology of gender difference in ED accesses for cardiovascular events.

Our data are not enough to derive conclusions, but, to our knowledge, this is the first report on gender difference in cardiovascular events after an earthquake and it may add a small chapter to the large literature on the complex pathophysiology and clinical aspects of cardiovascular disease in women.^13–22^

Unfortunately, there was no opportunity to carry out a survey on population surveillance, for lack of funds and of coordination between hospitals and territory. We have therefore only passively observed, in short and even more in the long term after the seismic event, an increase in risk factors mainly due to the change in lifestyle. These changes in lifestyle caused by the environmental disaster includeevacuation from houses, accomodations in common housings (tent cities) often for long periods of time, or placement in other more secure residences provided by families, friends, or local authorities. Earthquakes are “ideal” natural models also for chronic stress, with impact again on the cardiovascular system. Usually, to a first emergency phase of variable duration and related to the extent of the damages, it follows a reconstruction phase that takes much more time. At this stage, the media attention fades, but this is the most delicate period, in which the population that has suffered a disaster should not be abandoned because it is a long period. The lack of attention to a healthy lifestyle can result in the following years (perhaps even when it is finished rebuilding) an increase in morbidity/mortality or of precarious health. Reconstruction in fact is still ongoing. These sudden events have and are still producing little interest in many people to maintain or adopt healthy lifestyles: for example, people tend to smoke, former smokers started to smoke again. At the beginning choice of food was forced on the availability or sharing meals in the community, but now this is improved. Physical activity decreased because of security or inaccessibility of the sports facilities, which represented nonpriority in the reconstruction. In addition, there was abandonment of drug therapies, not only due to the difficulties at the time of seismic events to maintain prescriptions, but afterward to a sense of fatalism that intervenes, mainly in elderly, as a result of these disasters.

The responsibility of watching over the affected population's health status is therefore falling back on the sturdy shoulders of General Practitioners, who have worked hard and still are, but this probably is not enough. These considerations appear particularly important for our country, in the light of the recent earthquakes that struck and devastated central Italy with effect from August 24, 2016 continuing to create damage due to the earthquake swarm. The recent earthquakes and relative number of deaths (more than 300) have been much more severe than in our area in 2012 (fewer than 100 deaths), but, similarly, heavy damage occurred to rural buildings and mainly entire small villages were completely destroyed. Those villages lived exclusively on tourism; their historical monuments and churches do not exist any more and people did not accept to move from their houses to the hotels on the coast, but most were forced to move from ghost hamlets.

It is too early to analyze post-traumatic stress effects on cardiovascular events.

## Abbreviations Used

ACS: acute coronary syndrome
BP: blood pressure
DVT/PE: deep venous thrombosis and pulmonary embolism
ED: Emergency Department
HF: heart failure
NSTEMI: non-ST elevation myocardial infarction
SD: standard deviation
SNS: sympathetic nervous system
STEMI: ST elevation myocardial infarction
TIAs: transient ischemic attacks
UA: unstable angina
